# Study on the Geographic Variation and Trophic Variation of *Todarodes pacificus* in Different Areas Based on Stable Isotope Information from the Gladius

**DOI:** 10.3390/biology12040551

**Published:** 2023-04-04

**Authors:** Huajie Lu, Rui Wang, Jing Chen, Yuzhe Ou, Maolin Zhao, Biqiang Zhang

**Affiliations:** 1 College of Marine Sciences, Shanghai Ocean University, Shanghai 201306, China; 2 Key Laboratory of Marine Ecological Monitoring and Restoration Technologies, MNRs, Shanghai 201306, China; 3 National Distant-Water Fisheries Engineering Research Center, Shanghai Ocean University, Shanghai 201306, China; 4 Scientific Observing and Experimental Station of Oceanic Fishery Resources, Ministry of Agriculture and Rural Affairs, Shanghai 201306, China

**Keywords:** stable isotope, gladius, *Todarodes pacificus*, East China Sea, Sea of Japan, geographic variation, trophic variation

## Abstract

**Simple Summary:**

*Todarodes pacificus* is an important cephalopod. This study analyzed the migration path and feeding ecology of *T. pacificus* by the stable isotope values of the proostracum of the gladius. This result showed that *T. pacificus* in the East China Sea migrated to low latitudes and nearshore areas during their life history, and the trophic level of their food exhibited no large changes during migration. *T. pacificus* in the Sea of Japan migrated to high latitudes and offshore areas during their life history, and the trophic level of their food decreased during migration. When the proostracum grew to 120 mm from the distal end, *T. pacificus* in both areas began to migrate. This study provides a scientific basis for further study on the fishery ecology and life history of *T. pacificus*.

**Abstract:**

The Japanese flying squid (*Todarodes pacificus*) is an important cephalopod in the northwest Pacific Ocean. In this study, the proostracum of the gladius of *T. pacificus* samples collected by Chinese squid fishing vessels in the East China Sea and the Sea of Japan in August and December 2018 were continuously cut, and stable isotope values of the cut fragments were used to analyze the migration path and feeding ecology of *T. pacificus*. The results showed that when the proostracum grew to 120 mm from the distal end, *T. pacificus* began to migrate. In the East China Sea, *T. pacificus* migrated to low latitudes and nearshore areas, and the trophic level of their food showed no large changes during migration. In the Sea of Japan, *T. pacificus* migrated to high latitudes and offshore areas, and the trophic level of their food showed a decreasing trend during migration. There was no significant difference in migration or feeding ecology between females and males, but the competitive capacity of the females may be stronger than that of the males. The results provided a scientific basis for the scientific management and development of *T. pacificus* resources.

## 1. Introduction

The Japanese flying squid, *Todarodes pacificus*, is a warm–temperate oceanic cephalopod of the class Cephalopoda (Teuthoidea: Ommastrephidae) and genus *Todarodes*, which primarily inhabits the northwestern Pacific Ocean and the Gulf of Alaska in the eastern Pacific Ocean [1,2]. The distribution of *T. pacificus* is affected by ocean currents. The Sea of Japan, the Pacific coast of Japan, the East China Sea, and the Yellow Sea are all important fishing grounds for *T. pacificus* [3,4]. *T. pacificus* plays an important role in the marine ecosystem [5,6,7]. To date, national and international scholars have studied the resource distribution [5], age [8], population structure [9,10], and reproduction of *T. pacificus* [11,12]. Studies have shown that *T. pacificus* in the Sea of Japan migrates northward for feeding and then migrates southward for spawning, and the trophic level of feeding increases during migration [3,13], but the feeding ecology and migration path of *T. pacificus* in the East China Sea are less known.

Feeding analysis and stable isotope analysis (SIA) are the main research methods to analyze the feeding ecology and migration path of cephalopods [13]. Feeding analysis methods cannot reflect the long-term feeding habits of cephalopods, and this method only observes indigestible food, so the feeding analysis method has great uncertainty [14]. Stable isotope analysis can reflect the long-term and short-term feeding habits of cephalopods more quickly and objectively, so this method is widely used in studies on the feeding ecology and migration of cephalopods [15,16].

To date, national and international scholars have studied the biological characteristics of the hard tissues of cephalopods, such as beaks [17], statoliths [18], and gladii [19]. The gladius is a hard tissue composed of chitin and protein in the body of cephalopods, and the gladius records full information on the life history of cephalopods. The gladius is easy to obtain, preserve, and cut, so it is a good carrier to study the growth process of cephalopods [20,21,22]. In this study, the proostracum of the gladius of *T. pacificus* collected in the East China Sea and the Sea of Japan was cut, and then the carbon and nitrogen stable isotope ratios of the cut fragments were measured. Based on the stable isotopic values, this study analyzed the feeding ecology and migration path of *T. pacificus* in the East China Sea and the Sea of Japan.

## 2. Materials and Methods

### 2.1. Sampling

**Figure 1:**
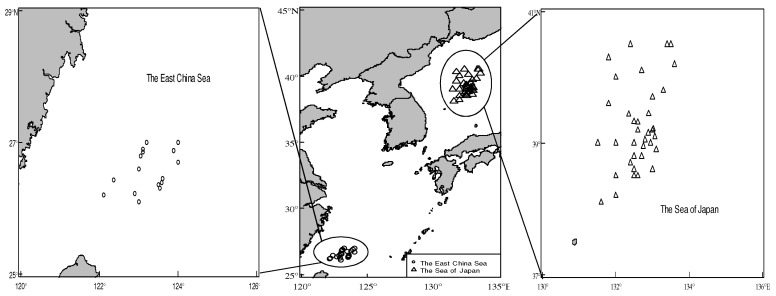
Distribution of sampling stations. (The circles represent sampling stations of *T. pacificus* in the East China Sea, the triangles represent sampling stations of *T. pacificus* in the Sea of Japan.)

A total of 955 samples was randomly sampled by Chinese squid fishing vessels in the southern part of the East China Sea and the Sea of Japan in August and December 2018 (Figure 1). All samples were immediately frozen and transported to the Biological Laboratory of Shanghai Ocean University. After thawing the samples in the laboratory, the fishery biological data were measured, including mantle length (ML) and body weight (BW), and the sex and maturity stage were identified through visual examinations. The mantle length was measured accurately to 0.1 cm, and the body weight was measured to 0.1 g. Gonad maturity consisted of stages 1–5 according to the cephalopod gonad maturity stage proposed by Ehrhardt [23]; those with gonad maturity in stages 1–2 were defined as immature individuals, and those with gonad maturity in stages 3–5 were defined as mature individuals [23,24]. Considering the sample size, sex ratio, sampling time, and other factors, 24 samples of *T. pacificus* were selected as the research objects, including 8 samples from the East China Sea and 16 samples from the Sea of Japan. The samples from the East China Sea were labeled G1, G2, G3…G8, and samples from the Sea of Japan were labeled G9, G10, G11, …, G24.

**Figure 2:**
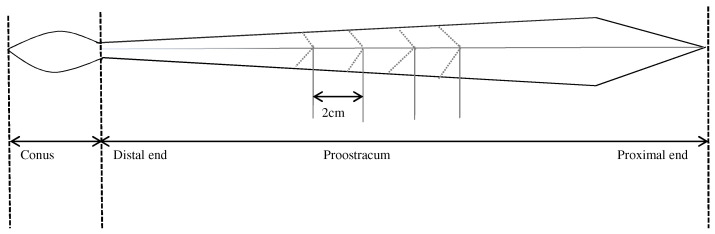
Structure of the *Todarodes pacificus* gladius.

The gladius of *T. pacificus* consists of 3 morphological parts: rostrum, conus, and proostracum. The gladius was extracted from the back of the mantle cavity and cleaned in an ultrasonic cleaner for 10 min to remove residual soft tissue. Based on the growth direction of the proostracum, from the distal end to the proximal end, the proostracum was consecutively cut every 2 cm with acetone-washed fine scissors following the ‘V’ shape of the growth lines (Figure 2). A total of 63 segments was cut from *T. pacificus* of the East China Sea, and a total of 155 segments was cut from *T. pacificus* of the Sea of Japan. All segments were rinsed again with distilled water, freeze-dried at −55 °C for ≥24 h, and homogenized into a fine powder using a Mixer mill MM440 (Retsch, Haan, Germany) prior to SIA [25,26].

### 2.2. Stable Isotope Analysis

A total of 0.1 mg of powder from each segment of proostracum among individuals was pooled using a tin capsule, and then the powder’s stable isotope ratios were measured using an IsoPrime isotope ratio mass spectrometer (IsoPrime Corporation, Cheadle, UK) and Vario Macro Elemental Analyzer (Elementar Analysensysteme GmbH, Hanau, Germany). The isotopic compositions of the samples were expressed as δ^13^C and δ^15^N using the equation

$$\delta X=[(R_{sample}/R_{standard})-1]\times 10^{3}$$

where *X* is ^13^C or ^15^N, and R_sample_ and R_standard_ are the corresponding ratios of ^13^C/^12^C or ^15^N/^14^N of the sample and the standard, respectively. To calibrate the system and compensate for drift, 3 standard items were placed for every 10 samples, and USGS 24 (−16.049‰v PDB) and USGS 26 (53.7‰v N_2_) were used to calibrate the δ^13^C and δ^15^N values, respectively. The analytical errors of δ^13^C and δ^15^N were approximately 0.05‰ and 0.06‰, respectively. Stable isotope analysis was conducted in the stable isotope laboratory of Shanghai Ocean University [25,26].

### 2.3. Statistical Analysis

Based on the stable isotopic values, this study drew a trophic niche plot and calculated the total area (TA) and standard ellipse corrected area (SEA_C_) of the East China Sea and the Sea of Japan. Linear regression analysis was used to analyze the relationships between the stable isotope values and the length of the gladius. A *t*-test was used to analyze the significant difference in the average δ^13^C and δ^15^N values, and the significance level was α = 0.05. This study used Excel (2010) and R Studio to plot the data and SPSS to analyze the data.

## 3. Results

### 3.1. Biological Parameters

In this study, the ranges of the ML and BW of the *T. pacificus* collected in the East China Sea were 18.7–23.0 cm and 142.4–270.7 g, respectively, and the ML and BW of the females were higher than those of the males (female: 22.0–23.0 cm and 240.3–270.7 g; male: 18.7–20.0 cm and 142.4–204.2 g).

The ranges of the ML and BW of the *T. pacificus* collected in the Sea of Japan were 22.0–25.8 cm and 275.3–377.2 g, respectively, and the ML and BW of the females and males were similar (female: 22.5–25.8 cm and 275.3–377.2 g; male: 22.0–25.5 cm and 279.3–372.1 g) (Table 1).

### 3.2. Stable Isotopic Values

For the *T. pacificus* collected in the East China Sea, the ranges of δ^13^C and δ^15^N values were −19.38‰ to −17.62‰ and 7.10 to 10.19‰, respectively. The stable isotope values of the females and males both increased gradually with the growth of the proostracum, and the stable isotopic values of the females were higher than those of the males. The δ^13^C and δ^15^N values of the females were −19.38‰ to −17.62‰ and 7.10‰ to 10.19‰, respectively. The δ^13^C and δ^15^N values of the males were −18.68‰ to −17.82‰ and 7.17‰ to 8.93‰, respectively.

For the *T. pacificus* collected in the Sea of Japan, the ranges of δ^13^C and δ^15^N values were −18.68 to −20.67‰ and 7.64 to 6.57‰, respectively. The stable isotope values of the females and males both decreased gradually with the growth of the proostracum, and the stable isotopic values of the females were similar to those of the males. The δ^13^C and δ^15^N values of the females were −20.67‰ to −18.68‰ and 6.59‰ to 7.64‰, respectively; the δ^13^C and δ^15^N values of the males were −20.53‰ to −18.61‰ and 6.57‰ to 7.74‰, respectively (Table 2).

### 3.3. Trophic Niche

**Figure 3:**
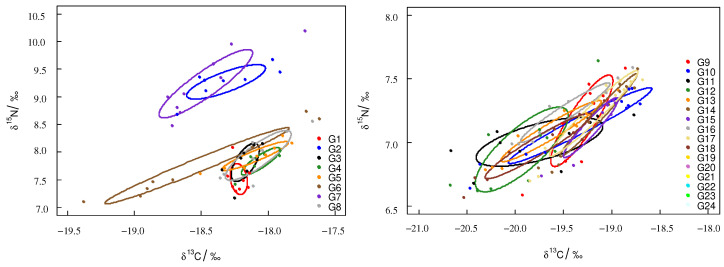
Trophic niche of *T. pacificus* collected in the East China Sea and the Sea of Japan (circle plot: SEAc).

According to the ontogenetic trophic niche research framework proposed by Hammerschlag-Peyer, the trophic niche plot was drawn using stable isotope values [27]. For *T. pacificus* collected in the East China Sea, the trophic niche area (SEAc) of the samples had significant differences, and the samples’ trophic niches did not overlap. Sample G6 had the largest SEAc. The trophic niches of G2 and G7 overlapped, and the trophic niches of G1, G3, G4, G5, and G8 overlapped. For *T. pacificus* collected in the Sea of Japan, the trophic niche areas (SEAc) of the samples were similar, and most samples’ trophic niches overlapped (Figure 3).

**Figure 4:**
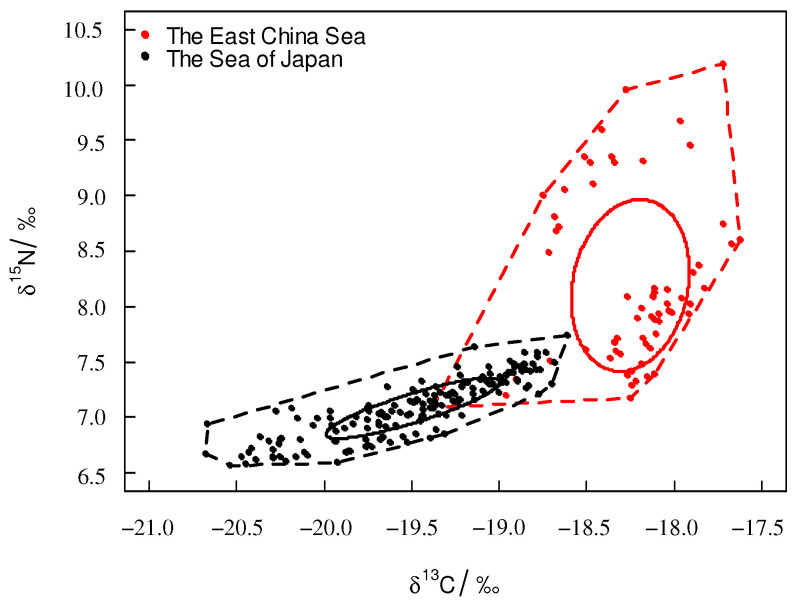
Trophic niche of *T. pacificus* collected in the different areas (square plot: TA; circle plot: SEAc).

For *T. pacificus* collected in the East China Sea, the TA and SEAc were 3.11‰^2^ and 0.810‰^2^, respectively. For *T. pacificus* collected in the Sea of Japan, the TA and SEAc were 1.21‰^2^ and 0.213‰^2^, respectively. The SEAc of the *T. pacificus* collected in the East China Sea was larger than that in the Sea of Japan. The trophic niche did not overlap between the two areas. There were significant differences between the trophic niches of the two areas (Figure 4).

**Figure 5:**
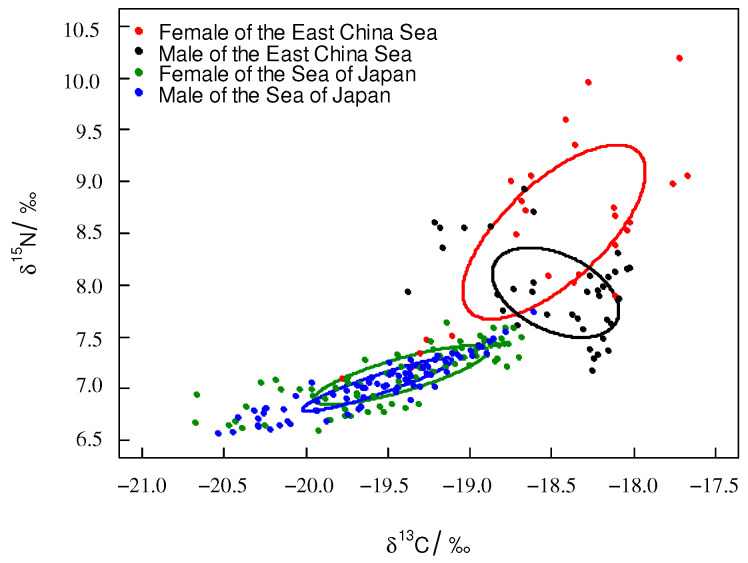
Trophic niche of *T. pacificus* of females and males collected in the different areas (circle plot: SEAc).

The trophic niche area (SEAc) of the females was larger than that of the males in both areas. For *T. pacificus* collected in the East China Sea, the SEAc of the females and males were 1.068‰^2^ and 0.48‰^2^, respectively. The overlapping SEAc was 0.239‰^2^ between females and males, and the overlapping rate was 18.3%. For *T. pacificus* collected in the Sea of Japan, the SEAc values of the females and males were 0.274‰^2^ and 0.139‰^2^, respectively. The overlapping SEAc was large between females and males, the overlapping SEAc was 0.126‰^2^, and the overlapping rate was 44.2% (Figure 5).

### 3.4. Geographic Variation and Trophic Variation

**Figure 6:**
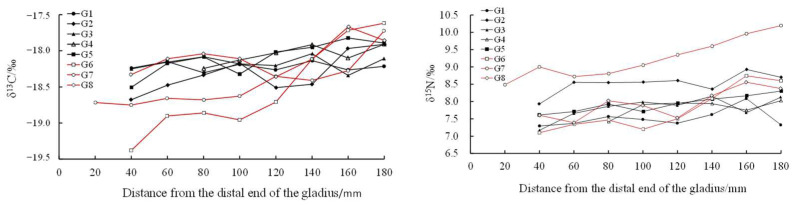
Relationship between δ^13^C‰, δ^15^N‰, and the growth of the proostracum of *T. pacificus* collected in the East China Sea. (Black line represents males, and red line represents females.)

**Figure 7:**
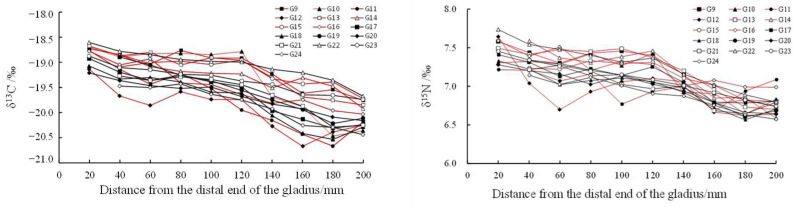
Relationship between δ^13^C‰, δ^15^N‰, and the growth of the proostracum of *T. pacificus* collected in the Sea of Japan. (Black line represents males, and red line represents females.)

With the growth of the proostracum, the stable isotope values showed a fluctuating trend. The stable isotope values of *T. pacificus* collected in the East China Sea increased gradually, but the stable isotope values of *T. pacificus* collected in the Sea of Japan decreased gradually. When the proostracum grew to 120 mm from the distal end, the δ^13^C and δ^15^N values of *T. pacificus* in the two areas began to change greatly (Figure 6 and Figure 7).

Linear regression analysis showed that for the *T. pacificus* collected in the East China Sea, there was a significant positive correlation between the average δ^13^C values and the growth of the proostracum (^13^C: r = 0.970, n = 9, *p* < 0.01), and there was no significant correlation between the average δ^15^N values and the growth of the proostracum (^15^N: r = 0.486, n = 9, *p* > 0.05). However, with the growth of the proostracum, the average δ^15^N values increased slightly. For the *T. pacificus* collected in the Sea of Japan, linear regression analysis showed that there was a significant negative correlation between the average δ^13^C values and the growth of the proostracum (^13^C: r = −0.977, n = 10, *p* < 0.01), and there was a significant negative correlation between the average δ^15^N values and the growth of the proostracum (^15^N: r = −0.961, n = 10, *p* < 0.01).

**Figure 8:**
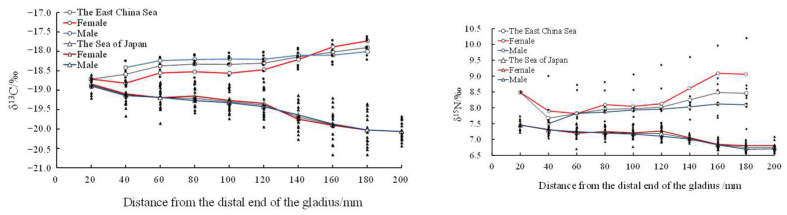
Relationship between average δ^13^C‰ and δ^15^N‰ values and the growth of the proostracum of *T. pacificus* collected in the East China Sea and the Sea of Japan. (Black line represents males, and red line represents females.)

There was no significant difference in the average δ^13^C (samples in the East China Sea, ^13^C: F = 0.003, n = 9, *p* = 0.977 > 0.05; samples in the Sea of Japan, ^13^C: F = 0.150, n = 10, *p* = 0.483 > 0.05) and δ^15^N (samples in the East China Sea, ^15^N: F = 2.640, n = 9, *p* = 0.063 > 0.0; samples in the Sea of Japan, ^15^N: F = 0.008, n = 10, *p* = 0.446 > 0.05) values in both the East China Sea and the Sea of Japan (Figure 8).

In addition, a *t*-test showed that there were significant differences in the stable isotopic values of *T. pacificus* between the East China Sea and the Sea of Japan (*t*-test, δ^13^C: F = 4.532, *p* = 0<0.01; δ^15^N: F = 1.164, *p* = 0<0.01). The stable isotopic values of *T. pacificus* in the East China Sea were significantly higher than those in the Sea of Japan (Figure 8).

## 4. Discussion

### 4.1. Trophic Niche

The trophic niche can reflect the trophic level of an organism in its ecosystem [16]. The relationship (overlap/independent) between trophic niche plots can reflect individuals’ or groups’ trophic niche relationships [27]. The trophic niche area can be used to analyze the competitive ability of individuals or groups; the larger the trophic niche area is, the stronger the competitive ability for resources [28]. In this study, for the *T. pacificus* samples collected in the East China Sea, the trophic niches of the G2, G6, and G7 samples did not overlap with those of the other samples, which may be because the G2, G6, and G7 samples came from different spawning grounds. The fact that there are multiple spawning grounds of *T. pacificus* in the East China Sea also supports this interpretation [29,30,31]. This phenomenon also occurred in other cephalopods. Gong [25] found that *Dosidicus gigas* samples collected from the coast of Peru had groups that came from different spawning grounds, and the trophic niches of samples that came from different spawning grounds did not overlap. The trophic niches of the G1, G3, G4, G5, and G8 samples overlapped with each other, indicating that these samples may come from the same spawning ground, and they have similar living environments. The migration paths and food of the samples were similar during their life history. Xie [32] found that *Sthenoteuthis oualaniensis* individuals with overlapping trophic niches have similar migration paths and food intake. In addition, the SEAc of the samples collected in the East China Sea were different, indicating that samples have different competitive abilities for resources. The different SEAc values may have been related to sex, mantle length, and living environment. The G6 sample had the largest SEAc, which may be related to the larger ML of G6. For the *T. pacificus* samples collected in the Sea of Japan, the trophic niches of the *T. pacificus* samples overlapped with each other, and the SEAc of the *T. pacificus* samples was similar, indicating that these samples came from the same spawning ground and preyed on food with similar trophic levels, and these samples had similar migration paths and feeding ecologies during their life history. The *T. pacificus* samples were collected in the northern part of the Sea of Japan, but the spawning ground of *T. pacificus* was mainly distributed in the southern part of the Sea of Japan [33]. Therefore, it is possible that the samples migrated northward during their life history.

This study analyzed the trophic niche between *T. pacificus* collected in the East China Sea and the Sea of Japan and found that the trophic niches of the two areas did not overlap. This result indicated that *T. pacificus* were spatially heterogeneous, so stable isotope values can be used to trace the different geographic regions of the *T. pacificus* group. Studies have shown that the different stable isotope values can trace the different groups of *D. gigas*, such as Chilean, Peruvian, and the central eastern Pacific [34]. In comparison to those in the Sea of Japan, *T. pacificus* samples collected in the East China Sea had a larger SEAc, which indicated that samples collected in the East China Sea occupied a higher position in the ecosystem. The trophic niches may have been related to factors such as latitude, feeding ecology, and living environment [35]. The larger SEAc of *T. pacificus* collected in the East China Sea was a result of the following factors: (1) The *T. pacificus* collected in the East China Sea had groups that came from different spawning grounds, so the *T. pacificus* collected in the East China Sea had a more complex feeding ecology and a larger SEAc. (2) The *T. pacificus* collected in the East China Sea had a higher stable isotope baseline. Studies have shown that there is a negative correlation between the stable isotopic baseline of phytoplankton and latitude, and the stable isotopic values decrease with increasing latitude [36]. This phenomenon also occurred in other cephalopods. For instance, Takai found that the δ^13^C value of *S. oualaniensis* muscle in low-latitude sea areas was higher than that in high-latitude sea areas [37]. The latitude of the East China Sea was lower than that of the Sea of Japan, so the *T. pacificus* collected in the East China Sea had a higher stable isotope baseline and a larger SEAc. (3) The *T. pacificus* collected in the East China Sea may prey on food at higher trophic levels. *T. pacificus* is an opportunistic predator, and it preys on different kinds of food in different areas [38]. Gong found that *D. gigas* in different areas prey on different kinds of food [34]. Currently, there are few studies on the feeding of *T. pacificus*, and the types of food consumed by *T. pacificus* in the East China Sea and the Sea of Japan are not clear. Further analysis can be carried out in combination with stomach content analysis.

In this study, for *T. pacificus* collected in the East China Sea and the Sea of Japan, there was significant overlap between the females and males. This result indicated that females and males had similar feeding ecologies and that there was strong competition between females and males. The trophic niche area of the females was larger than that of the males, which may indicate that females have a stronger competitive capacity than males. Females’ stronger competitive capacity may be caused by the fact that females prefer to prey on food of higher trophic level or larger size. Within the same population, females and males can reduce mutual competition and maintain functional status by distributing different habitats and food [39,40]. Females had higher energy requirements for individual and gonad development. Fang found that females of *Ommastrephes bartramii* have larger feeding organs, higher energy demands, and stronger feeding abilities [41]. This phenomenon also occurred in *D. gigas* [34]. Further analysis should be carried out in combination with a morphological analysis of feeding organs.

### 4.2. Geographic Variation and Trophic Variation

Stable isotope values in biological tissues can provide transitional information about matter and energy in food webs [21]. The value of δ^13^C (^13^C/^12^C) changes little with increasing trophic level, so δ^13^C can reflect the habitat of organisms and is mainly used to analyze the migration path of organisms. The value of δ^15^N (^15^N/^14^N) gradually increases with increasing trophic level, so δ^15^N can reflect the trophic level of organisms in an ecosystem, and δ^15^N can be used to analyze the feeding ecology of organisms [42].

The gladius is the hard tissue of cephalopods. Cephalopods can convert nutrients from food into chitin and protein, and then the chitin and protein form the new structure of the gladius. The growth of the gladius is irreversible and continues through the whole life history [21], so the gladius is a good carrier for recording life history information [14]. In this study, the δ^13^C and δ^15^N values of gladii were used to analyze the migration path and feeding ecology of *T. pacificus*. For the *T. pacificus* collected in the East China Sea, the δ^13^C and δ^15^N values of most samples increased with the growth of the proostracum. For the *T. pacificus* collected in the Sea of Japan, the δ^13^C and δ^15^N values of most samples decreased with the growth of the proostracum.

#### 4.2.1. Geographic Variation

Cephalopods have obvious migratory behaviors [38]. Water temperature, light intensity, CO_2_, and other conditions vary in different areas, so the δ^13^C values vary in different areas, and the variation in δ^13^C values can reflect the geographic variation in organisms. In this study, the stable isotope values of *T. pacificus* in the East China Sea and the Sea of Japan fluctuated with the growth of the proostracum of the gladius, and the fluctuations were different among individuals of the two areas, indicating that the *T. pacificus* of the two areas have heterogeneity. This phenomenon has also occurred in other cephalopods, such as *D. gigas* [34], *O. bartramii* [43], and *Sthenoteuthis pteropus* [44].

In this study, the δ^13^C values in the East China Sea and the Sea of Japan began to change significantly when the proostracum grew to 120 mm from the distal end, indicating that migration occurred when the proostracum grew to 120 mm from the distal end. In this study, for *T. pacificus* samples collected in the East China Sea, although the samples may come from different spawning grounds in the East China Sea, there was a significant positive correlation between the δ^13^C values and the growth of the proostracum. The δ^13^C values were related to latitude. The higher the latitude was, the lower the δ^13^C value was [36]. In addition, the value of δ^13^C was also related to the distance from the shore. The nearshore sea area has sufficient light, continental runoff, and various nutrients, making the nearshore sea area productive and nutritious and having higher δ^13^C values [14]. Ruiz-Cooley et al. [45] found that the δ^13^C and δ^15^N values of oceanic cephalopod muscles had no fixed variation trend with latitude and mantle length, but the δ^13^C and δ^15^N values decreased with increasing offshore distance. Based on the above factors, it can be inferred that *T. pacificus* collected in the East China Sea gradually migrated to low-latitude and nearshore areas during its life history. For *T. pacificus* samples collected in the Sea of Japan, there was a significant negative correlation between the δ^13^C values and the growth of the proostracum, indicating that these samples migrated to high-latitude and offshore areas during their life history. Some studies have found that *T. pacificus* migrates northward to feed along both sides of the Japanese archipelago [38], which is consistent with the results of this study. In this study, for *T. pacificus* collected in the East China Sea and the Sea of Japan, there was no significant difference in δ^13^C values between females and males, indicating that there was no difference in migration paths between females and males. However, as the gladius grows, it lengthens and thickens, so the previous gladius may contain the growth increments of the subsequent long-term history. This phenomenon may influence the accuracy of the δ^13^C values and the migration path prediction, so further studies could be carried out in combination with stable isotopic values of biological tissues.

The migration path was greatly different between *T. pacificus* collected in the East China Sea and the Sea of Japan, and this phenomenon may have been caused by the fact that the two areas contain different populations of *T. pacificus*. Studies have shown that different populations have certain differences in morphology, spawning grounds, migration paths, and other characteristics [38]. According to molecular biological techniques, the results of the unweighted pair-group method with arithmetic means (UPGMA) showed that the population structure of *T. pacificus* mainly consisted of two clades, the autumn clade and the nonautumn clade [46]. This population division was mainly caused by geographical isolation [38], which was also consistent with the geographical differences in this study. Further studies could, thus, be carried out in combination with population division information.

#### 4.2.2. Trophic Variation

The δ^15^N value is affected by the stable isotope baseline and feeding. The variation in the δ^15^N value can be used to analyze the trophic level of the feeding [42]. Lorstrain inferred the approximate period of the feeding transition of *D. gigas* based on the change in the δ^15^N value of the gladius [47]. In this study, *T. pacificus* collected in both the East China Sea and the Sea of Japan began to migrate when the proostracum grew to 120 mm from the distal end, and the δ^15^N value also began to change at this time. The variation in the δ^15^N value may be due to the following two reasons: One reason was migration in different areas with different stable isotopic baselines. Another reason was the changes in feeding during migration. In this study, for *T. pacificus* collected in the East China Sea, there was no correlation between the δ^15^N value and the growth of the proostracum. The δ^15^N values of most *T. pacificus* individuals did not significantly increase, indicating that the food trophic level of most individuals did not significantly change during migration. Ruiz-Cooley found that with the growth of *D. gigas*, the δ^15^N value of the gladius gradually increases, and the trophic level of the food prey increases significantly [48]. Some studies have also shown that the trophic level of the food preyed upon by *T. pacificus* will gradually increase with body growth [49]. However, this study’s result was contradictory to the above results, and the result of this study may be caused by La Niña events during the life history of *T. pacificus* collected in the East China Sea. According to the ONI index, La Niña events occurred in the spring of 2018. Some studies have shown that large-scale environmental changes can affect the migration and feeding of cephalopods [30]. The occurrence of La Niña was not conducive to the growth of *T. pacificus* and led to a reduction in the suitable spawning ground area and the abundance of the community resources of *T. pacificus* [50]. Ning also found that during the La Niña of 2018, the feeding stage of *T. pacificus* was lower and mainly in stages 0–2 [51]. Therefore, the occurrence of La Niña events may reduce the bait resources of *T. pacificus*, so the food trophic level of most *T. pacificus* individuals did not change. Further studies could be carried out in combination with the change in sea surface temperature (SST) caused by La Niña events.

For *T. pacificus* collected in the Sea of Japan, there was a significant negative correlation between the δ^15^N value and the growth of the proostracum. This indicates that the food trophic level of *T. pacificus* individuals decreased gradually during migration, which may be caused by the decrease in the trophic level of the food preyed upon by *T. pacificus* or the increase in the proportion of low-trophic level food preyed upon by *T. pacificus* during migration. Alegre [52] and Gong [34] also found that the food trophic level of *D. gigas* decreases during migration. *T. pacificus* is an opportunistic predator with no fixed feeding strategy, and it eats different kinds of food in different areas. Studies have shown that the main stomach contents of *T. pacificus* collected in the Sea of Japan in winter were small fish, but other studies have shown that mature *T. pacificus* mainly prey on fish and cephalopods [34]. Therefore, in this study, the decreased δ^15^N values of *T. pacificus* may be caused by the proportion of small fish being eaten increasing and the proportion of larger fish and cephalopods being eaten decreasing during migration. However, as mentioned above, as the gladius grows, it lengthens and thickens, so the previous gladius may contain the growth increments of the subsequent long-term history. This phenomenon may influence the accuracy of the δ^15^N values and the trophic variation prediction, so further studies could be carried out in combination with stable isotopic values of biological tissues. For *T. pacificus* collected in the East China Sea and the Sea of Japan, there was no significant difference in δ^15^N values between the females and males (*p* > 0.05), indicating that the trophic levels of food were similar between females and males.

## 5. Conclusions

In this study, the proostracum of *T. pacificus* was cut, and the stable isotope ratio of the cut fragments was measured. Based on the trophic niche and stable isotope ratio sequence, this study analyzed the geographic and trophic variations in *T. pacificus* collected in the southern part of the East China Sea and the Sea of Japan and analyzed the reasons for the difference in the geographic and trophic variations in *T. pacificus* in the two areas. The study showed that in the East China Sea, *T. pacificus* migrated to low latitudes and nearshore areas, and the trophic level of *T. pacificus* food showed no great changes during migration. In the Sea of Japan, *T. pacificus* migrated to high latitudes and offshore areas, and the trophic level of *T. pacificus* food showed a decreasing trend during migration. *T. pacificus* began to migrate when the proostracum grew to approximately 120 mm from the distal end. There was no significant difference in migration and feeding ecology between females and males, but the competitive capacity of the female group may be stronger than that of the male group. Future studies can combine age information, stomach content analysis, muscle stable isotope information, and environmental data to analyze the biological characteristics of *T. pacificus*.

## Figures and Tables

**Table 1:** Biological parameters of *T. pacificus* samples.

No.	Sampling Location	Body Weight (g)	Mantle Length (mm)	Sampling Data	Sex	Maturity Stage
G1	123°58′ E, 26°39′ N	188.0	200.4	9 July 2018	M	4
G2	123°58′ E, 6°39′ N	204.2	200.5	9 July 2018	M	4
G3	123°58′ E, 26°39′ N	168.3	195.3	9 July 2018	M	4
G4	123°58′ E, 26°39′ N	142.4	187.2	11 July 2018	M	3
G5	123°58′ E, 26°39′ N	185.5	199.0	11 July 2018	M	4
G6	123°58′ E, 26°39′ N	240.3	230.1	11 July 2018	F	1
G7	123°58′ E, 26°39′ N	270.7	225.0	25 July 2018	F	4
G8	123°58′ E, 26°39′ N	254.4	220.0	25 July 2018	F	1
G9	133°07′ E, 39°10′ N	293.3	235.2	11 December 2018	F	1
G10	133°07′ E, 39°10′ N	377.2	258.4	11 December 2018	F	4
G11	133°07′ E, 39°10′ N	348.5	240.3	11 December 2018	F	4
G12	132°35′ E, 39°44′ N	373.3	250.3	9 December 2018	F	4
G13	132°35′ E, 39°44′ N	350.6	240.1	9 December 2018	F	4
G14	132°35′ E, 39°44′ N	290.4	235.1	9 December 2018	F	1
G15	132°35′ E, 39°44′ N	275.3	225.0	9 December 2018	F	3
G16	131°25′ E, 38°25′ N	290.5	230.0	15 December 2018	F	3
G17	131°25′ E, 38°25′ N	285.2	220.3	15 November 2018	M	3
G18	131°25′ E, 38°25′ N	295.7	234.4	15 November 2018	M	3
G19	131°25′ E, 38°25′ N	297.4	235.4	15 November 2018	M	3
G20	131°25′ E, 38°25′ N	372.1	255.4	15 November 2018	M	4
G21	133°25′ E, 40°25′ N	290.3	235.5	20 December 2018	M	3
G22	133°25′ E, 40°25′ N	350.0	250.2	20 December 2018	M	4
G23	133°25′ E, 40°25′ N	340.0	244.1	20 December 2018	M	4
G24	133°25′ E, 40°25′ N	279.3	225.6	20 December 2018	M	2

**Table 2:** Stable isotope values of the gladius of *T. pacificus*.

No.	Number of Segments of Proostracum	δ^13^C (‰)				δ^15^N (‰)			
		Mean	SD	Maximum	Minimum	Mean	SD	Maximum	Minimum
G1	8	−18.22	0.06	−18.14	−18.30	7.52	0.26	8.08	7.29
G2	8	−18.31	0.27	−17.91	−18.68	8.52	0.29	8.93	7.93
G3	8	−18.17	0.10	−18.04	−18.34	7.82	0.32	8.15	7.17
G4	6	−18.05	0.13	−17.91	−18.24	7.83	0.22	8.03	7.42
G5	8	−18.10	0.23	−17.82	−18.51	7.93	0.24	8.30	7.61
G6	8	−18.53	0.64	−17.62	−19.38	7.75	0.64	8.74	7.10
G7	9	−18.47	0.33	−17.72	−18.75	9.24	0.58	10.19	8.48
G8	8	−18.07	0.23	−17.67	−18.36	7.94	0.42	9.06	7.89
G9	10	−19.14	0.38	−18.73	−19.76	7.22	0.31	7.58	6.77
G10	10	−19.33	0.70	−18.70	−20.47	7.13	0.28	7.42	6.64
G11	10	−19.75	0.62	−18.77	−20.67	7.01	0.18	7.22	6.68
G12	10	−19.93	0.45	−19.14	−20.67	6.95	0.31	7.64	6.62
G13	10	−19.19	0.43	−18.68	−19.85	7.23	0.31	7.49	6.70
G14	9	−19.31	0.30	−18.86	−19.92	7.17	0.34	7.59	6.59
G15	9	−19.22	0.27	−18.97	−19.73	7.12	0.22	7.33	6.74
G16	10	−19.48	0.44	−18.78	−20.03	7.24	0.22	7.59	6.99
G17	10	−19.63	0.49	−18.93	−20.31	7.09	0.23	7.41	6.78
G18	10	−19.76	0.52	−19.08	−20.53	6.97	0.24	7.33	6.57
G19	9	−19.57	0.47	−18.90	−20.22	7.01	0.26	7.34	6.60
G20	10	−19.67	0.32	−19.21	−20.16	6.99	0.22	7.28	6.65
G21	9	−19.64	0.41	−19.27	−20.29	6.98	0.23	7.28	6.64
G22	10	−19.05	0.31	−18.61	−19.68	7.27	0.30	7.74	6.80
G23	10	−19.35	0.28	−18.88	−19.74	7.09	0.23	7.45	6.74
G24	9	−19.86	0.39	−19.43	−20.44	6.89	0.20	7.14	6.57

## Data Availability

The datasets used and/or analyzed during the current study are available from the corresponding author upon reasonable request.
